# Soluble transforming growth factor beta-1 enhances murine mast cell release of Interleukin 6 in IgE-independent and Interleukin 13 in IgE-dependent settings *in vitro*

**DOI:** 10.1371/journal.pone.0207704

**Published:** 2018-11-16

**Authors:** David O. Lyons, Michele R. Plewes, Nicholas A. Pullen

**Affiliations:** 1 School of Biological Sciences, University of Northern Colorado, Greeley, Colorado, United States of America; 2 Olson Center for Women's Health, Department of Obstetrics and Gynecology, University of Nebraska Medical Center, Omaha, Nebraska, United States of America; King's College London, UNITED KINGDOM

## Abstract

**Introduction:**

For immune cells transforming growth factor beta-1 (TGF-β1) can enhance or repress effector functions. Here, we characterize the effects of TGF-β1 on IgE-mediated and IL-33-mediated activation of primary murine mast cells derived from hematopoietic stem cells (bone marrow derived mast cells; BMMC). We also investigated potential interactions between TGF-β1 and stem cell factor (SCF). We conclude TGF-β1 plays a selectively stimulatory role for mast cell cultures *in vitro*.

**Methods:**

BMMCs from C57BL/6 mice were differentiated with IL-3 and then treated with TGF-β1. BMMCs were exposed to TGF-β1, primed with IgE, activated with antigen, and then IL-6 and IL-13 cytokine release was quantified using ELISA. Additionally, the effects of TGF-β1 on both IgE and IL-33-mediated short term activation were observed via flow cytometric analysis of both surface LAMP-1 expression and intracellular IL-6. Receptor colocalization was visualized using fluorescence confocal microscopy and individual receptor expression levels were also quantified.

**Results:**

Resting IL-6 production increased with TGF-β1 but significance was lost following BMMC activation via IgE receptor (FcεRI) crosslinking. This was similar to a comparison effect due to SCF treatment alone, which also enhanced resting levels of IL-6. TGF-β1 treatment enhanced release of IL-13 only with FcεRI-IgE-mediated activation. TGF-β1 suppressed mobilization of IL-6 with short-term BMMC activation when stimulated with IL-33. Lastly, colocalization patterns of the SCF receptor (CD117) and FcεRI with IgE crosslinking were unaffected by TGF-β1 treatment, but individual expression levels for FcεRI, CD117, and TGFβRII were all reduced following either IgE activation or TGF-β1 treatment; this reduction was partially recovered in BMMCs that were both activated by IgE and treated with TGF-β1.

**Discussion:**

These data reveal a novel positive effect of soluble TGF-β1 on mast cell activation *in vitro*, suggesting mast cells may be activated through a non-canonical pathway by TGF-β1. Understanding this interaction will provide insight into the potential role of mast cells in settings where TGF-β1 is produced in an aberrant manner, such as in and around high grade tumors.

## Introduction

Transforming Growth Factor Beta (TGF)-β1 is a widely expressed cytokine. The TGF-β1 signaling pathway evolved approximately one billion years ago as an immune regulatory mechanism among vertebrates. TGF-β1 modulates cellular responses starting with binding to TGF-β receptor II (TGFβRII). TGFβRII then aggregates with TGF-βRI at the cell surface inducing the phosphorylation of Smad proteins intracellularly. Ultimately, this cascade reaches the nucleus for transcriptional regulation. TGF-β1 is documented as generally inhibitory starting in the 1990s: as an anti-inflammatory, anti-autoimmune cytokine [1].

Mast cells are myeloid lineage cells of hematopoietic origin. They are present in the skin and along mucosal membranes, especially the gut where they combat helminth parasites. Well known for their roles in allergic pathologies, mast cells are broadly key to physiology at mucosal barriers. Mast cells are capable of collecting and presenting antigen to other cells and can also be induced to express MHC class II [2,3]. They are central in driving a T_H_2 response, inducing B cells to class switch to IgE via IL-4 and IL-13 [4]. Canonical activation of mast cells starts with the priming of their high affinity IgE receptor, FcεRI; in the body, mast cells are stably coated with IgE bound to FcεRI. Upon multivalent antigen binding of these IgE-FcεRI complexes the receptors cluster–crosslink (XL)–and internalize, triggering signaling cascades resulting in degranulation of the cell and activation of transcription factors, such as STAT5, to upregulate cytokine production [4]. Hours to days later these cytokines (*e*.*g*., IL-6, IL-13) are secreted [2]. They also exhibit important alternative pathways to activation, such as through IL-33 and its receptor (ST2), independent of FcεRI [5]. While the downstream effects of IL-33-mediated activation are comparable to IgE-mediated activation, the signaling pathways and mediators involved differ and there is evidence of extracellular parasites targeting this specific pathway to dampen anti-worm responses [6]. IL-33 treatment has also been found to enhance IgE-mediated activation by both increasing the number of degranulating cells in addition to enhancing the amount of chemokine production from activated cells [7]. Mast cells can also be activated through additional pathways by a variety of IgE/FcεRI mediators such as other Fc receptors and Toll-like receptors [8].

Recent evidence suggests that the interaction between mast cells and typical immunosuppressive cytokines varies unlike other immune cells and may be context dependent. A typical inhibitory cytokine, IL-10, was shown to behave as an immunostimulant when given to mast cells and in the development of mucosal food allergies [9]. At the post-transcriptional level, IL-10 was found to regulate microRNAs which enhanced skin mast cell secretion of IL-6 and IL-13 [10]. These interactions necessitate closer examination of the behavior of mast cells regarding common stimulatory and inhibitory molecules. Herein, we describe effects of TGF-β1 on IgE-mediated mast cell activation by observing the production of two cytokines commonly associated with this process: IL-6 (a broadly inflammatory cytokine) and IL-13 (a chemotactic cytokine that drives a T_H_2 response). The cells used in this study were grown without stem cell factor (SCF) unless explicitly noted for comparison. Treatment with SCF, although beneficial for BMMC proliferation *in vitro*, has been suggested to diminish IgE-mediated cytokine production and degranulation in BMMCs grown in SCF for four weeks [11]. These recent findings also apply to our understanding of TGF-β1 as well, warranting future research into context-specific TGF-β1 signaling.

Furthermore, studies suggest that TGF-β1 can also play a stimulatory role on IL-13 production. Patients with varying conditions of bone marrow fibrosis were found to have higher numbers of mast cells and mast cell infiltration along with increased levels of IL-13 and TGF-β1; however the direct sources of these cytokines were not shown [12]. Others have shown that regulatory T cells (T_reg_) enhance mast cell IL-6 production but strictly through TGF-β1 attached to the T_reg_ cell surface [13]. Thus, we posit a direct stimulatory effect of soluble TGF-β1 on mast cells, and perhaps other cells of the myeloid lineage, which would be a potent expansion from cell-restricted TGF-β1. This study specifically confirms the positive effects of TGF-β1 on resting mast cell production of IL-6; we demonstrate that soluble (not cell restricted) TGF-β1 amplifies mast cell release of this cytokine independent of IgE-mediated activation. Additionally, we show TGF-β1 treatment enhances IL-13 production upon IgE-mediated activation of BMMCs; conversely, TGF-β1 exhibited a suppressive effect on IL-33-mediated activation in agreement with previous work in the literature [14]. In conjunction, mast cells were observed through confocal fluorescence microscopy to identify potential cross-talk between the TGF-β receptor family and the definitive mast cell functional receptors c-kit (SCF receptor, CD117) and FcεRI. TGF-β1 did not affect the aggregation of these various receptors and to our knowledge this is the first report detailing this in the context of IgE-mediated activation of mast cells.

## Materials and methods

### Mice

C57BL/6 mice were housed and humanely euthanized by CO_2_ inhalation to effect in accordance with protocol #1702C-NP-M-20, which was approved by the University of Northern Colorado Institutional Animal Care and Use Committee. When possible, tissue was obtained from control mice scheduled for other experiments.

### BMMC differentiation

BMMC were cultured in RPMI 1640 (Thermo Fisher) supplemented to: 10% FBS (VWR), 1% pen/strep (Thermo Fisher), 2 mM L-Glutamine (Thermo Fisher), 1 mM Sodium Pyruvate (Thermo Fisher), 10 mM HEPES (Thermo Fisher), 30 ng/mL recombinant murine IL-3 (Peprotech), and 0.05 mM β-mercaptoethanol (Bio-Rad). Bone marrow was flushed from mouse femurs and tibias using unsupplemented RPMI then cleared of erythrocytes with ACK lysis buffer (Quality Biological). Cultures were initiated at over 500,000 cells/mL in T75 flasks (VWR); individual mouse cultures were kept separate. Cultures were observed daily; if adherent cells (~50% confluency) were present, cells were transferred to a new flask. Cultures were maintained between 250,000 and 1,000,000 cells/mL for 4–6 weeks to achieve pure BMMC populations. Successful differentiation was confirmed through flow cytometry (FcεRI^+^/c-kit^+^) prior to use in experiments using an Alexa Fluor 647-conjugated FcεRI antibody (Biolegend, #134309) and a FITC-conjugated CD117 antibody (Biolegend, #105805) per manufacturer recommendations. Samples were analyzed for phenotyping using a Sony SH800S Cell Sorter and independently validated with an Attune NxT cytometer; all data were processed in FCS Express (De Novo Software).

All cultures (n = 4–6 biological replicates) were randomly divided among treatment groups. When applicable for comparison to a known stimulant of resting BMMC, SCF-treated cell populations were supplemented with 10 ng/mL recombinant murine SCF (Peprotech).

### TGF-β1 treatment

For TGF-β1-treated groups, SCF-treated and untreated cells were distributed in 6-well plates at a density between 250,000 and 1,000,000 cells/mL. Appropriate groups were treated with 2 ng/mL recombinant murine TGF-β1 (Cell Signaling Technologies) and maintained for 48 hours before IgE priming to ensure cells were treated for a total of 72 hours prior to experimentation (timing includes IgE-mediated activation).

### IgE-mediated activation and quantification of BMMC cytokines

Cells (either grown in the presence or absence of SCF) were incubated overnight with 0.5 μg/mL of IgE (BD Biosciences, #557079) specific to trinitrophenyl keyhole limpet hemocyanin (TNP-KLH; Santa Cruz Biotechnologies). Treatments were rinsed with unsupplemented RPMI and resuspended in complete medium. The IgE-FcεRI complexes were crosslinked by adding 0.3 μg/mL TNP-KLH and incubating overnight (IgE-exposed, no TNP-KLH controls for each group were run separately). Supernatants were collected the following day and stored at -80°C for later use. ELISAs were performed on conditioned media according to the manufacturer’s protocols for IL-6 (PeproTech; 900-T50) and IL-13 (PeproTech; 900-K207). The effects of SCF were analyzed in these data as a comparison treatment to observe apparent differences in mast cell function.

### LAMP-1 degranulation assay

Transport of Lysosomal-associated membrane protein 1 (LAMP-1) to the surface of the cell is a viable indicator of mast cell degranulation during IgE-mediated activation [15]. Cells were evenly distributed in 24-well plates at densities between 250,000 and 1,000,000 cells/mL. Appropriate groups were treated with TGF-β1 as described for 72 hours prior to activation. For IgE-mediated activation, cells were treated with IgE after 48 hours of TGF-β1 treatment and left to incubate overnight. Cells were then washed with 1x PBS and resuspended in complete RPMI without IL-3 and left to incubate at 37°C for 2–3 hours. Cells were then blocked with Fc Block (anti-mouse CD16/32 antibody; Biolegend, #101302) for 10 minutes according to manufacturer’s recommendation and then stained with APC-conjugated CD107a (LAMP-1) antibody (Biolegend, #121613) at the manufacturer’s recommended dilution and left to incubate at 37°C for 1 hour. Cells were then activated with TNP-KLH as described previously and set to incubate again for 10 minutes at 37°C. Cells were then centrifuged and resuspended in flow cytometry buffer (0.5% BSA in Ca^2+^/Mg^2+^ free 1x Dulbecco’s Phosphate Buffered Saline, ThermoFisher). Samples were analyzed using a Sony SH800S Cell Sorter and independently validated with an Attune NxT cytometer; all data were processed in FCS Express (De Novo Software).

### Intracellular flow cytometry

Cells were evenly distributed in 24-well plates at densities between 250,000 and 1,000,000 cells/mL and treated with TGF-β1. Appropriate groups were treated with TGF-β1 as described above for 72 hours prior to activation. Appropriate groups were then activated with either 0.3 μg/mL TNP-KLH or 50 ng/mL of recombinant murine IL-33 (Peprotech) for 90 minutes. All groups were then treated with 1x monensin solution for 4–6 hours (BioLegend). Cells were then centrifuged and resuspended in 4% paraformaldehyde and incubated for 20 minutes in the dark. Cells were then centrifuged and resuspended in permeabilization buffer (0.1% Saponin, BioLegend); this step was repeated two additional times. Following this, suspensions were aspirated so as to leave 100 μL of fluid in the tube. Appropriate groups received PE-conjugated IL-6 antibody (BioLegend, #504504) per the manufacturer’s recommendation and left to incubate in the dark for 20–30 minutes. Finally, cells were washed twice with permeabilization buffer and resuspended in a final volume of 500 μL of flow cytometry buffer. Samples were analyzed using a Sony SH800S Cell Sorter and independently validated with an Attune NxT cytometer; all data were processed in FCS Express (De Novo Software).

### Microscopy

Cells were treated (TFG-β1 or IgE-XL) as stated previously. Following treatment, cells were rinsed three times with 1x PBS, fixed with 4% paraformaldehyde for 30 minutes at 4°C, rinsed again and blocked (5% normal goat serum).

Primary antibodies (FITC-CD117 (mouse-mAB; 1:50; Biolegend, #105805), Alexa Fluor 647-FcεR1 (mouse-mAB; 1:50; Biolegend, #134309), and TGFβRII (rabbit-mAB; 1:50; ABClonal #A11765)) were incubated at 4°C for 24 h. Following incubation, cells were rinsed three times with 1x PBS and centrifuged at 500 g for 5 minutes to remove unbound antibody. Anti-rabbit Alexa Fluor 568 (1:500; Thermo Fisher, #A11011) was then added and left to incubate for 1 hour at room temperature. Cells were mounted onto a glass microscope slide using 10 μL SlowFade-Gold DAPI mounting medium (Thermo Fisher) and No. 1 cover slip.

Images were collected using a Zeiss confocal microscope equipped with a 100x oil objective (N.A. = 1.4), an acquisition image size 512x512 pixels (33.3 μm x 33.3 μm), and light collection at 450 nm—1000 nm. Thirty cells were randomly selected from each slide for analysis. For all microscopy analysis z-stack images were converted into maximum intensity projections and processed utilizing ImageJ (National Institutes of Health) analysis software. For colocalization analysis, the JACoP plug-in was used to determine the Manders’ coefficient for each image as described in [16]. Manders’ M1 coefficient ranged from 0 to 1, multiplied by 100%. Total cell fluorescence intensity of each receptor was determined for individual cells as previously described [16–18].

### Statistics

Cytokine secretion measured by ELISA was analyzed in pg/mL normalized per 10^6^ cells in triplicate for four to six biological replicates. Data are presented as the mean±SEM with significance at p<0.05. LAMP-1 and intracellular cytokine expression were run in triplicate and these data are also presented as the mean±SEM.

Fluorescence microscopy data are reported as least square means±SEM with significance at p<0.05. The effects of TGF-β1 and FcεRI crosslinking on colocalization of BMMC surface receptors were analyzed using one-way ANOVA.

All multiple comparisons data were tested for normality using the Shapiro-Wilk test then analyzed using a one-way ANOVA with Tukey’s multiple comparisons test. All analyses were conducted using Prism 7 (GraphPad).

## Results

### BMMC culture and phenotyping

**Fig 1:**
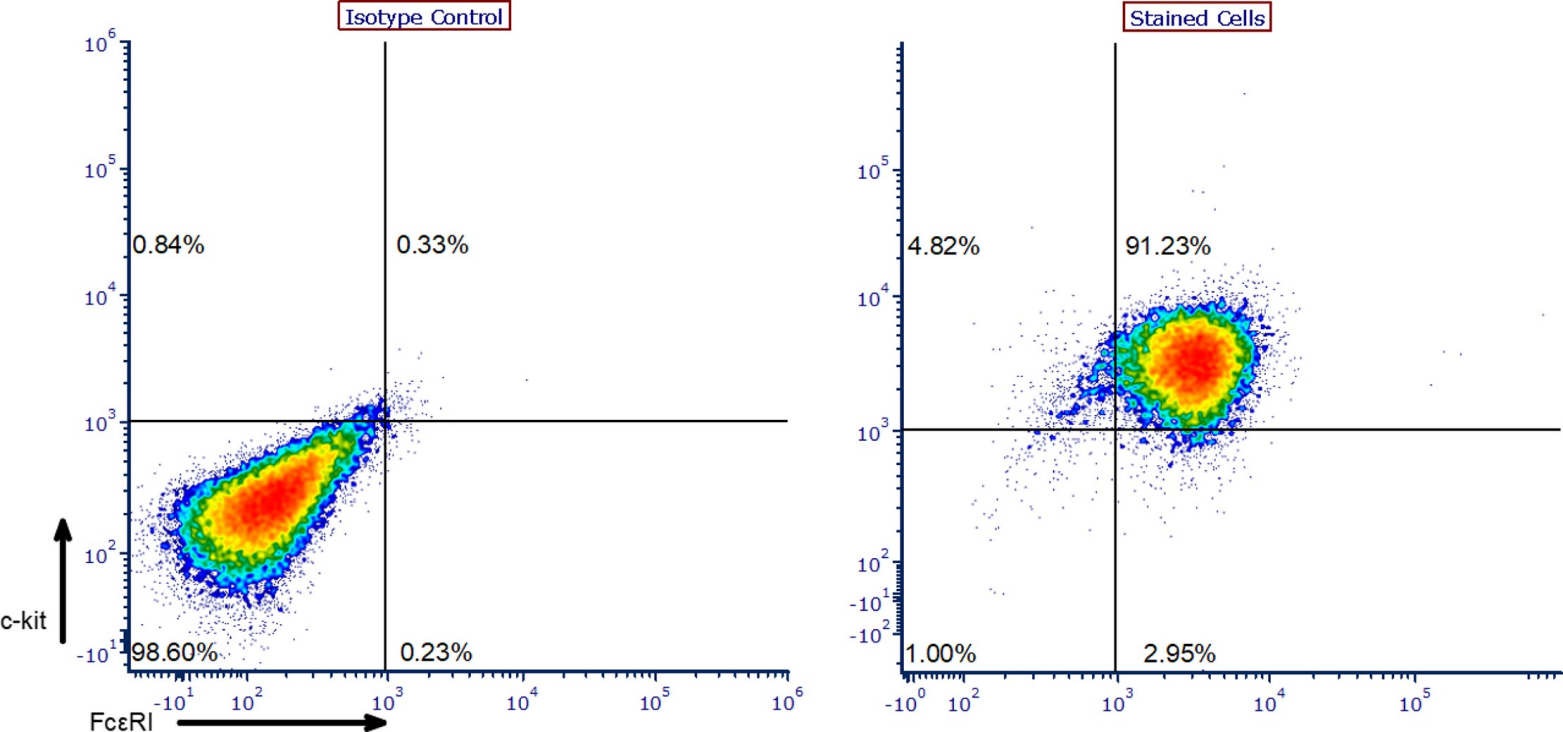
BMMC phenotyping. Representative data of BMMC phenotyping results. Cells were stained for CD117 (c-kit; FITC-conjugated) and FcεRI (Alexa Fluor 647-conjugated) to confirm successful differentiation.

BMMCs were harvested from mice as previously described. Harvested cells were cultured for at least four weeks prior to usage in experiments in complete RPMI supplemented with 30 ng/mL recombinant murine IL-3. BMMCs were phenotyped using flow cytometry to confirm successful differentiation through detection of both FcεRI and CD117 (c-kit) on the cell surface in over 90% of the population (Fig 1).

### TGF-β1 enhances IL-6 secretion in resting BMMCs and enhances IL-13 secretion only from IgE-activated BMMCs

**Fig 2:**
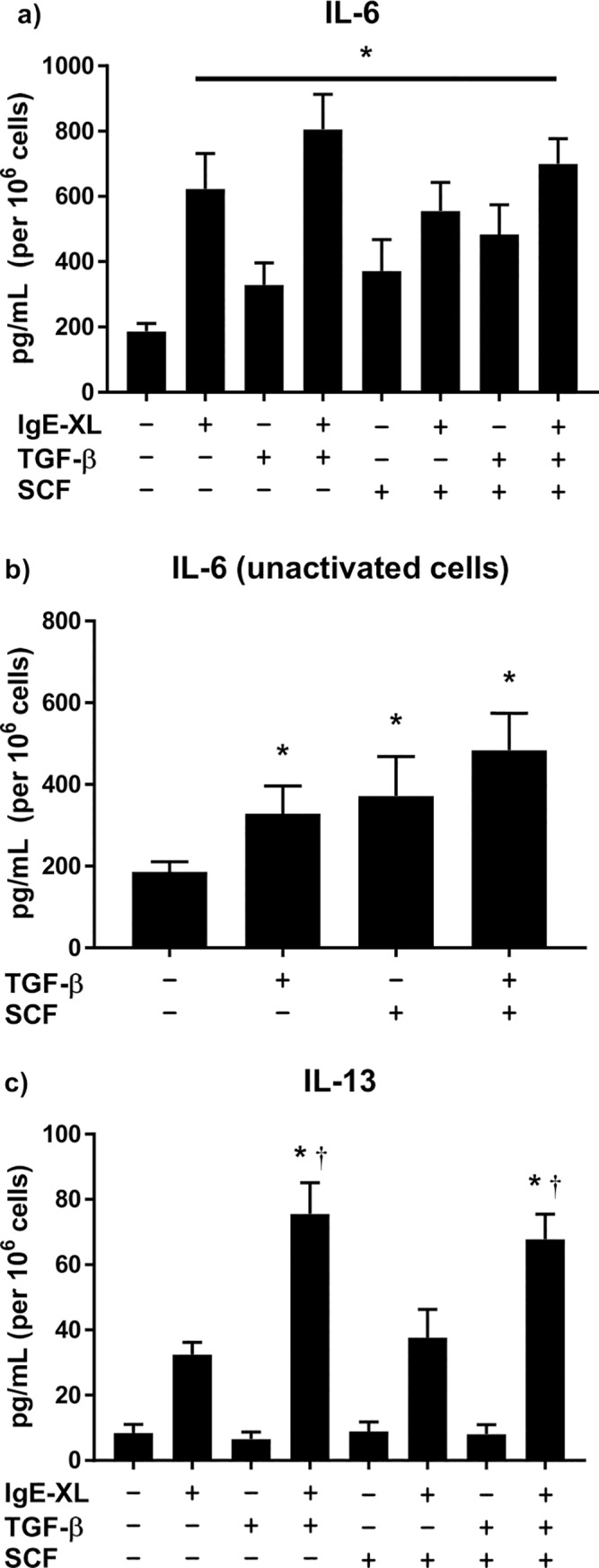
IL-6 and IL-13 secretion by mast cells based on treatment. Raw cytokine production for IL-6 **(a)** and IL-13 **(c)** normalized as secretion per 10^6^ cells. **(b)** IL-6 production in resting (no IgE-crosslink) BMMCs only (emphasis from 2a above). * in **(a)** and **(b)** indicates significant difference from no IgE-crosslink, no TGF-β1, no SCF control. * in **(c)** indicates significant difference from IgE-crosslinked, no TGF-β1, no SCF control; † indicates significant difference from IgE-crosslinked, no TGF-β1, SCF-treated control. Significance was noted at p<0.05.

BMMCs were treated with TGF-β1, SCF (for a resting comparison), or both and then activated by crosslinking FcεRI. IL-6 was increased from a mean of 186 pg/mL per 10^6^ cells to about 622 pg/mL per 10^6^ cells following IgE-mediated activation (Fig 2A) but IL-6 was also increased in cells treated with either SCF or TGF-β1 alone (371 and 328 pg/mL per 10^6^ cells respectively), independent of IgE-mediated activation; cells treated with both cytokines exhibited 483 pg/mL per 10^6^ cells prior to activation and 699 pg/mL per 10^6^ cells following activation (Fig 2A and 2B). Unlike IL-6, IL-13 secretion was only enhanced in IgE-activated cells. TGF-β1 treatment increased IL-13 secretion in activated cells both with SCF (67 pg/mL per 10^6^ cells) and without SCF treatment (75 pg/mL per 10^6^ cells) compared to the untreated control (32 pg/mL per 10^6^ cells) (Fig 2C). Treatment with SCF alone caused no significant difference in IL-13 secretion from activated BMMCs when compared to untreated cells. All resting BMMCs secreted comparable (8–10 pg/mL per 10^6^ cells) levels of IL-13 regardless of treatment. These data show that soluble TGF-β1 directly enhances mast cell IL-6 production independent of IgE-mediated activation. TGF-β1 was also sufficient in enhancing IL-13 secretion in activated BMMCs but had no effect in resting BMMCs. Overall, TGF-β1 appears to modulate late phase BMMC activation by enhancing cytokine secretion of both IL-6 and IL-13; enhanced IL-6 secretion was also observable without IgE-crosslinking.

### TGF-β1 enhances short-term IgE-stimulated surface LAMP-1 recruitment but not intracellular IL-6

**Fig 3:**
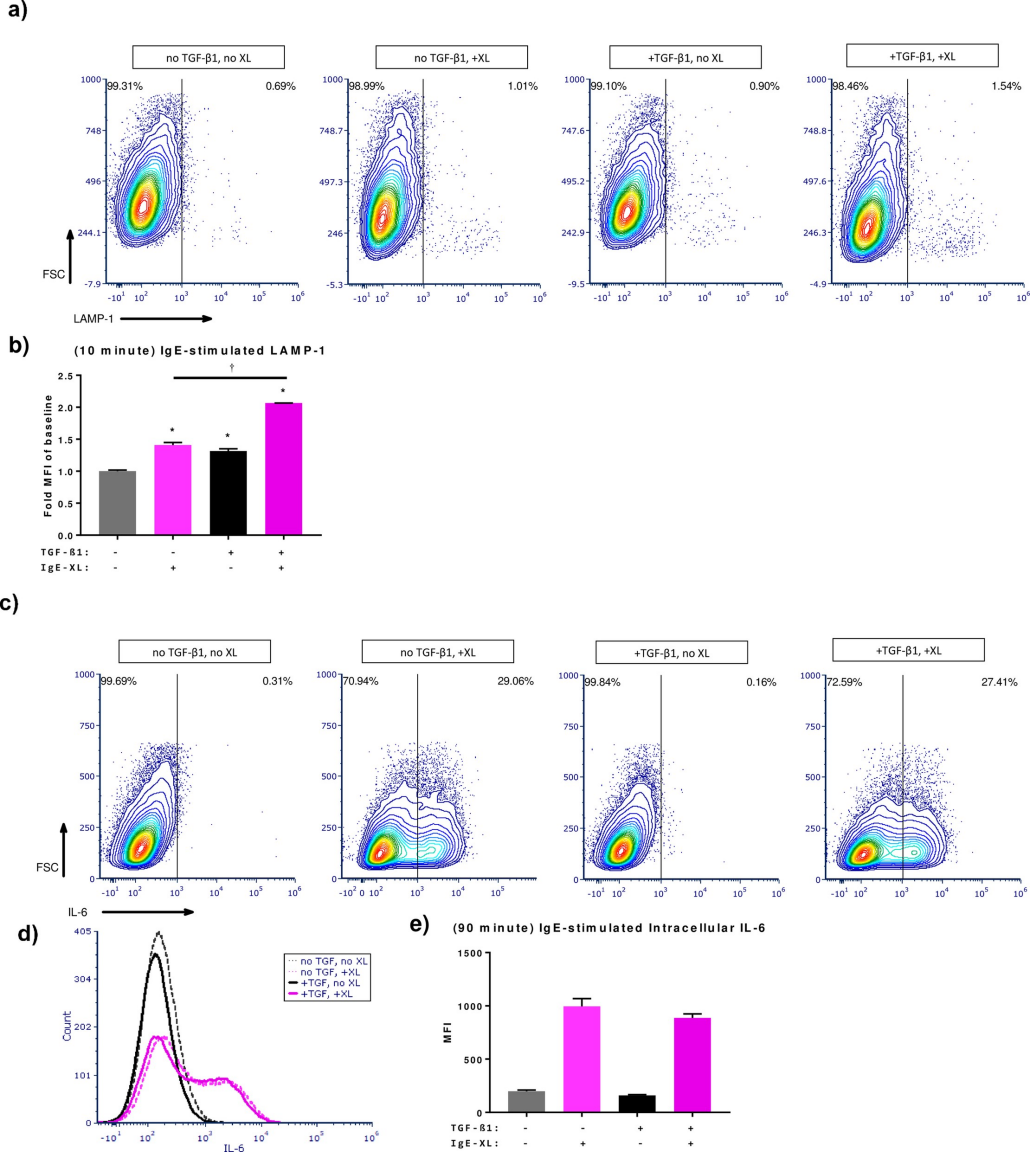
IgE-stimulated LAMP-1 translocation and early phase IL-6 production. Representative cytograms of **(a)** surface LAMP-1 expression (10 minute activation) and **(c)** intracellular IL-6 (90 minute activation) analyzed via flow cytometry. **(b)** Fold change of mean fluorescence intensity compared to baseline (no TGF-β1, no IgE crosslink) for LAMP-1 expression; * indicates significant difference from baseline, † indicates significant difference compared to TGF-β1-treated, IgE-crosslinked groups. **(d)** Representative histogram of intracellular IL-6. **(e)** Mean fluorescence intensity for intracellular IL-6. Significance was noted at p<0.05.

To evaluate the effects of TGF-β1 on BMMC degranulation, an early-phase activation response, surface LAMP-1 expression was measured following BMMC stimulation via IgE [15]. BMMCs were pre-treated with TGF-β1 72 hours prior to experimentation. Surface LAMP-1 (10 minute activation) and intracellular IL-6 (90 minute activation) expression were visualized using flow cytometry. Treatment with TGF-β1 was sufficient in enhancing modest but significant LAMP-1 expression independent of IgE-mediated activation (Fig 3A and 3B). In cells stimulated with TNP-KLH TGF-β1 further enhanced surface LAMP-1 expression. This effect is apparent in both the flow cytograms as well as the fold change in mean fluorescence intensity (MFI) compared to the no TGF-β1, no IgE crosslink group (Fig 3B). While intracellular IL-6 expression was found to trend lower with TGF-β1 treatment in activated BMMCs this difference was not significant (Fig 3C–3E). These data show a stimulatory effect of TGF-β1 treatment on a surrogate of very early BMMC activation (LAMP-1) but no significant difference in early phase intracellular IL-6 recruitment.

### TGF-β1 inhibits IL-33-incuded intracellular IL-6

**Fig 4:**
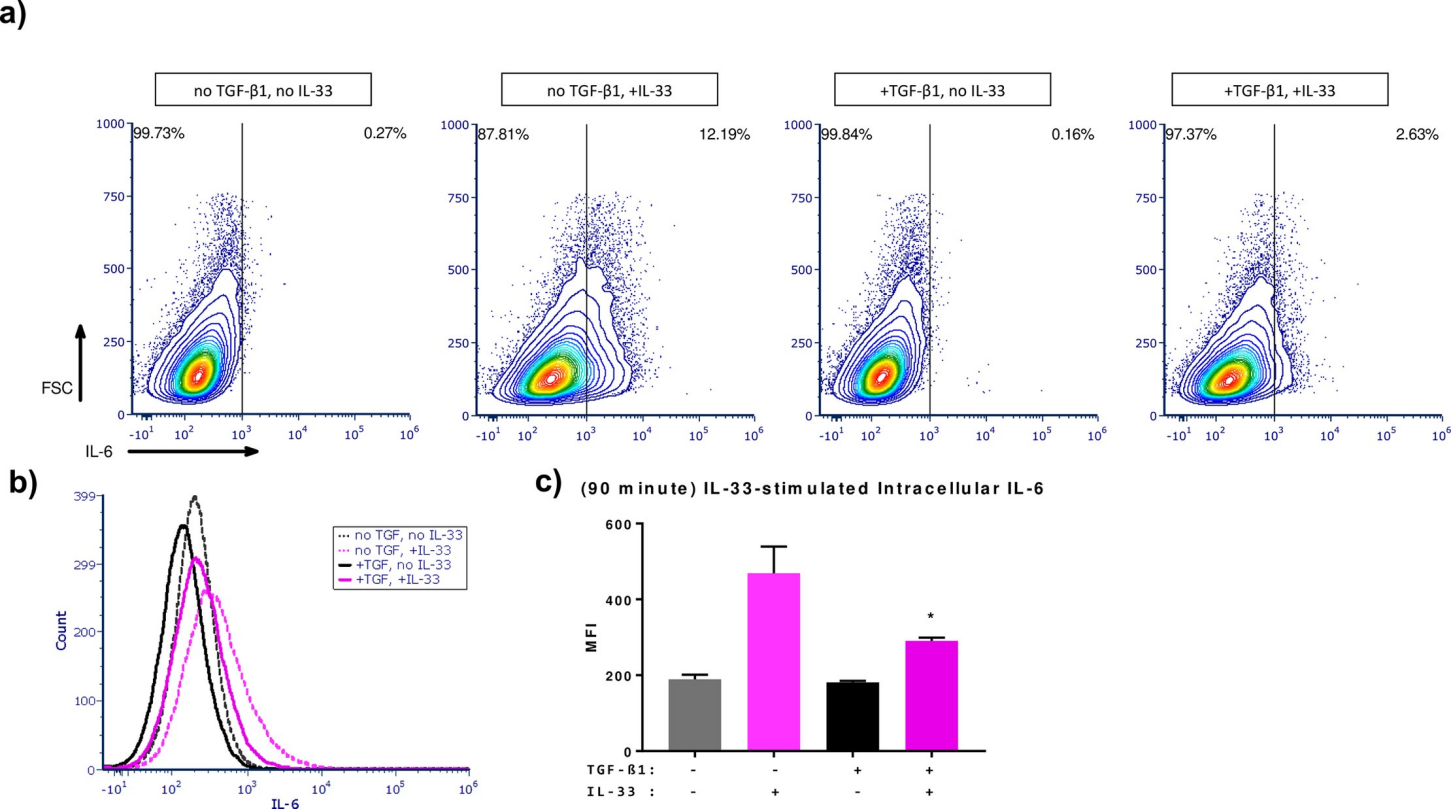
IL-33-stimulated early phase IL-6 production. **(a)** Representative cytograms of intracellular IL-6 (90 minute activation) analyzed via flow cytometry. **(b)** Representative histogram of intracellular IL-6. **(c)** Mean fluorescence intensity for intracellular IL-6; * indicates significant difference (p<0.05) from no TGF-β1, IL-33-activated control.

To assess the effects of TGF-β1 on alternative pathways of mast cell activation BMMCs were pre-treated with TGF-β1 72 hours prior to experimentation as described in the methods. BMMCs were briefly activated with IL-33 and intracellular IL-6 expression (90 minute activation) was visualized using flow cytometry. Intracellular IL-6 was notably reduced in TGF-β1 treated IL-33-activated BMMCs compared to IL-33 activation alone (Fig 4A–4C). This finding supports previous data that showed TGF-β1 having a suppressive effect on IL-33 mediated cytokine secretion [14].

### IgE crosslinking decreases FcεRI-CD117 (c-kit) percent colocalization independent of TGF-β1 treatment

**Fig 5:**
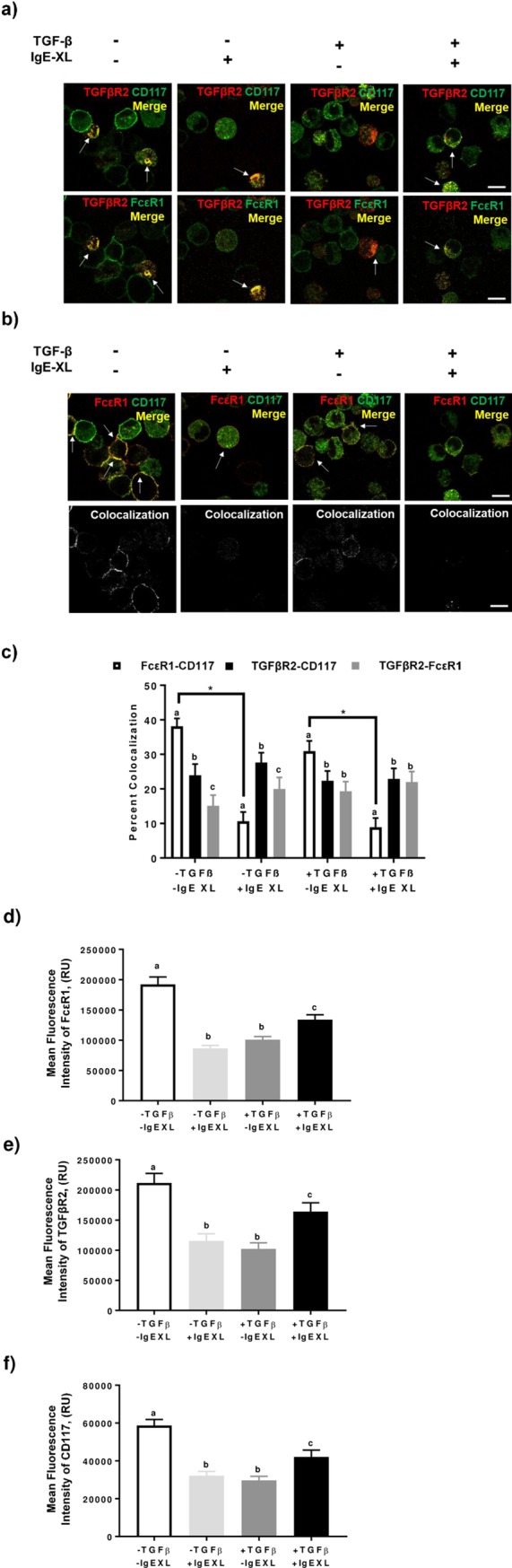
Colocalization of TGFβRII, FcεRI, and CD117. **(a)** Representative micrographs of surface expression of TFG-βRII with FcεRI or CD117. **(b)** Representative micrographs of FcεRI and CD117 surface expression; corresponding colocalization shown below. Arrows represent regions of colocalization. Micron bar represents 10 μm. **(c)** Quantitative analyses of colocalization within treatment. Means with different letters^abc^ differ significantly *within* treatment (p<0.05) and lines with * indicate significant differences *between* treatments. **(d-f)** Mean fluorescence intensities for individual surface expression of FcεRI **(d)**, TGFβRII **(e)**, and CD117 **(f).** Means with different letters^abc^ differ significantly.

To attempt to characterize the mechanism behind TGF-β1’s stimulatory effects on mast cell activition at the cell surface, percent colocalization of FcεRI-CD117, TGFβRII-CD117, and TGFβRII-FcεRI were determined using immunofluorescence microscopy (Fig 5). Colocalization of TGFβRII with canonical markers of mast cells, CD117/c-kit and FcεRI, was not found to be significantly different across treatment groups (Fig 5A and 5C). IgE crosslinking (IgE-XL) of BMMCs decreased percent colocalization of FcεRI and CD117 in both the presence and absence of TGF-β1; TGF-β1 treatment was also sufficient in reducing colocalization of these receptors prior to activation, however this was not significant when compared to the untreated group (Fig 5B and 5C). Additionally, the decreased percent colocalization of FcεRI and CD117 following IgE-crosslinking was proportional with and without TGF-β1 treatment, indicating TGF-β1 does not directly affect this surface receptor colocalization shift on BMMCs (Fig 5C). Colocalization data for other combinations of receptors are provided in S1 Fig.

### TGF-β1 and/or IgE crosslinking are sufficient in reducing surface FcεRI, TGFβRII, and CD117 expression

BMMCs were pretreated with TGF-β1 as described in the methods. The individual mean fluorescence intensities for each receptor were quantified. Treatment with TGF-β1 significantly reduced surface expression of all three receptors compared to untreated control (Fig 5D–5F). In addition, activation of BMMCs with TNP-KLH resulted in a comparable reduction in surface expression. Interestingly, TGF-β1 treatment in conjunction with IgE-crosslinking mitigated the reduction of surface expression of all three receptors; this partial recovery was significantly higher than the single treatments but also significantly lower than untreated, unactivated BMMCs (Fig 5D–5F).

## Discussion

In our experiments, soluble TGF-β1 stimulated IL-6 secretion independent of IgE-mediated activation. TGF-β1 has been shown to promote mast cell IL-6 production in the context of lung inflammation; this promotes neutrophil apoptosis and clearance [19]. However, this effect involved T_reg_ cell surface sequestered TGF-β1 [13]. Our findings suggest that TGF-β1 plays a directly stimulatory role on IL-6 production, independent of IgE status and potentially independent of immunosuppressive T_reg_, which in turn could elicit acute phase inflammatory responses. Characterizing the mechanism(s) involved and carefully testing the direct effect(s) of soluble TGF-β1 on other myeloid cells is warranted. Alternatively, myeloid-derived suppressor cells (MDSC) utilize IL-6 to support tumor progression through production of TGF-β1 [20,21]. MDSCs also enhance IL-6 and IL-13 secretion by activated mast cells [22]. Carefully studying the interactions between TGF-β1, IL-6, T_reg_, MDSC, and mast cells will be insightful in chronic inflammatory settings such as some high-grade solid cancers. In other pathologies, such as systemic sclerosis, TGF-β (including isoforms 1 and 2) was detectable within the mast cells of both diseased and healthy human patients; diseased patients exhibited a higher amount within their mast cells and in the extracellular space [23]. The ability for mast cells to both produce and respond to TGF-β suggests possible autocrine and paracrine signaling that could occur and result in the development of chronic inflammation and other pathologic conditions. In mice, chronic treatment with high doses of TGF-β1 resulted in inflammation of the tongue, esophagus, and skin. Additionally, increased levels of TGF-β1 were localized in these locations and were detectable in the saliva [24]. These findings show a pathological phenotype resulting from excess TGF-β1 signaling, coinciding with our results regarding late phase cytokine secretion by BMMCs even without IgE-mediated activation of the cells.

Here, TGF-β1 also enhanced production of IL-13 following mast cell activation via FcεRI. IL-13 is a major cytokine in T_H_2-related immune responses likely responsible for the clearing of large extracellular insults such as gut parasites [25]. However, unmitigated IL-13 release will drive B-lymphocyte class switching to IgE, which in turn coats naïve mast cells via FcεRI, thus prompting a vicious T_H_2 cycle [26]. Our experiments show that murine TGF-β1 directly enhances IgE-mediated IL-13 production in murine BMMC which propagates such pathologies. This suggests that TGF-β1 enhances T_H_2 skewing in allergic responses and could contribute to hypersensitivity in individuals prone to allergic disease. Observing this effect in mice prone to allergies (such as Balb/c mice) will prove insightful. A possible push toward T_H_2-like cytokine production by TGF-β1 was first reported in a mouse T lymphoma model but such effects could be very context-dependent [27].

In the present study, IgE-mediated short-term activation as measured by LAMP-1 translocation was significantly higher in BMMCs treated with TGF-β1 in both resting and activated treatments. Short-term intracellular IL-6 recruitment was comparable in resting BMMCs regardless of TGF-β1 treatment; IgE-activated BMMC groups (+/- TGF-β1) were not different in their expression however IgE-activated BMMCs treated with TGF-β1 were trending downward. This trend is contradictory to secretion data presented in Fig 2, suggesting that TGF-β1 might exhibit a similar stimulatory effect to IL-33 on IgE-mediated activation by enhancing late phase cytokine production only through a long term genomic expression effect. IL-33 has been shown to enhance IgE-mediated food anaphylactic responses in mice through enhanced cytokine production as well as enhanced IgE-mediated degranulation through surface LAMP-1 expression. Our LAMP-1 data depict a similar effect on IgE-stimulated LAMP-1 expression with TGF-β1 [28]. Furthermore, the apparent lack of enhancement in our intracellular data compared to the long-term secretion data could be due to the shorter window of activation and suggests that TGF-β1 may modulate immediate and late phase responses differently in mast cells.

In terms of IL-33-mediated activation, intracellular IL-6 expression was found to be inhibited by TGF-β1 in IL-33-activated BMMCs, confirming previous findings [14]. This suppression of intracellular IL-6 expression contradicts our IgE-mediated IL-6 secretion data however this could be due again to the duration of activation between the two experiments and/or the fact that IgE- and IL-33-mediated activation, while likely having some transduction overlap, are separable cell signaling events [7]. TGF-β1 appears to modulate immediate and late phase responses differently. Therefore, measuring intracellular cytokine expression at various time points would help explain this observed difference.

Through fluorescence microscopy we observed potential effects of TGF-β1 and one of its receptors, TGFβRII, at the signaling apex of the definitive mast cell surface receptors FcεRI and CD117 (SCF receptor). FcεRI and CD117 colocalization is lost upon crosslinking of FcεRI with IgE-antigen complexes. This is not surprising as FcεRI internalization following cross-linking is known [29]. However, this decline in colocalization is unchanged with TGF-β1 treatment, suggesting there is no apparent surface cross-talk occurring among these receptors that is dependent on the presence of TGF-β1. It has been shown that TGF-β1 transcriptionally represses FcεRI and CD117 through regulation of Etf homologous factor (Ehf) [30]. Single channel mean fluorescence intensities for each receptor confirm that TGF-β1 does indeed inhibit receptor expression of FcεRI, CD117, and TGFβRII, aligning with previous findings described in Gomez et al. as well as Kashyap et al. [31,32]. Nonetheless, this is discordant with other work reporting suppression of CD117 only and not FcεRI [33]. Treatment with just TGF-β1 or crosslinking alone resulted in comparable declines of expression for all three receptors. However, treatment with both TGF-β1 and IgE-crosslinking resulted in a partial recovery of expression for all three receptors. These observations suggest that the activation of both the TGF-β and IgE pathways may enhance receptor transport to the cell surface as demonstrated by the increased LAMP-1 surface expression in IgE-activated BMMCs treated with TGF-β1. In regards to human pathology, the TGF-β pathway has been shown to be activated in response to an allergen challenge in conjunction with activin signaling pathways. Expression of TGFβRII was found to be significantly enhanced in atopic patients 24 hours after antigen challenging [34]. This finding contradicts another study using human mast cells which found no expression of the endothelial TGF-β type 1 receptor, ALK-1, following treatment with any TGF-β isoform however this could be due to different durations of treatment [35]. TGF-β1 and activin signaling have been shown to lead to the expansion of T_regs_ however enhanced TGF-β1 signaling in the presence of IL-6 also promotes differentiation of T_H_17 cells which leads to chronic pathologies [36,37]. Proteomic/transcriptomic pathway analysis of TGF-β1 engaging in cross-pathway signaling such as activin would elucidate the multifarious effects of TGF-β1 on immune cell function.

The microscopy data presented depict a partial recovery in TGFβRII with concomitant IgE crosslinking, suggesting that TGF-β1 treatment initially results in reduced TGFβRII due to receptor internalization. Interestingly, a similar effect was observed for all three receptors; these receptors may reside on lipid rafts along the cell and are internalized simultaneously. TGFβRII has been observed between microdomains of the plasma membrane in both non-raft clathrin-coated pits as well as caveolin-1 positive, cholesterol-rich lipid rafts and the signaling potential of this receptor can vary depending on its localization [38]. Further research into the internalization of this receptor and its intracellular processing in this context is warranted along with research into activin signaling in BMMCs.

In sum, we demonstrate that soluble TGF-β1 plays a context-dependent, direct stimulatory role on primary mast cells *in vitro*. The specific molecular mechanism underlying this effect is still unknown. TGF-β1 might modulate targets outside of the canonical TGF-β1 signaling cascade (Smads) however further research into this is necessary. IL-6 secretion was increased independent of IgE-mediated activation, suggesting that TGF-β1 non-canonically targets the MAP kinase or Akt pathways to enhance IL-6 production [39]. Future research into the phosphorylation patterns of this pathway will reveal the specific effects of TGF-β1. Additionally, IL-13 production was altered only after IgE activation, suggesting that TGF-β1 can modulate the FcεRI signaling pathway *(e*.*g*., STAT5) in a stimulatory manner. Altogether these data necessitate careful further examination of soluble TGF-β1 with respect to mast cell effector functions.

## Limitations

The data presented in this study focus on observing the functional effects of TGF-β1 treatment on primary murine derived BMMCs. We study these mast cells for the advantages of using primary cells over immortalized cell lines, which often necessitate carrying mutations that obfuscate signaling relevant to TGF-β1; however there are some limitations to our design. For these experiments, IL-6 and IL-13 secretion data were used to illustrate the effect of TGF-β1 on late phase mast cell activation; early phase responses were measured through surface LAMP-1 expression and intracellular IL-6 mobilization. Additional assays of immediate mast cell activation (e.g. β-hexosaminidase assay or cysteinyl leukotriene release assay) are necessary to confirm the effect of TGF-β1 on degranulation. These experiments were all performed *in vitro*, suggesting some of the results depicted here might not translate into an observable effect *in vivo*. In addition, only BMMCs were assessed in this manuscript; adult mucosal, connective tissue, and peritoneal mast cells were not analyzed. The application of these findings to human pathologies was not directly tested and future research into the effects of TGF-β1 on human derived mast cells would help to confirm the results presented here. Lastly, the findings presented here do not evaluate changes in gene expression or specific mechanisms/pathways (MAPK, Akt, STAT5, as have been partially studied by Pullen [40] and Fernando [41]) involved in the effects presented–additional large scale proteomics and/or RNA sequencing will provide insight into the underlying signaling networks responsive to TGF-β1 treatment on mast cell function.

## Supporting information

S1 Fig Representative single channel fluorescence and colocalizations for TGFβRII, CD117, and FcεRI under conditions of +/- IgE-XL and +/- TGF-β1. (TIF)
