# Supplementary figures and images for: Soluble transforming growth factor beta-1 enhances murine mast cell release of Interleukin 6 in IgE-independent and Interleukin 13 in IgE-dependent settings in vitro
Source: PLoS One. 2018 Nov 16;13(11):e0207704. doi: 10.1371/journal.pone.0207704 (PMC6239331; doi:10.1371/journal.pone.0207704)

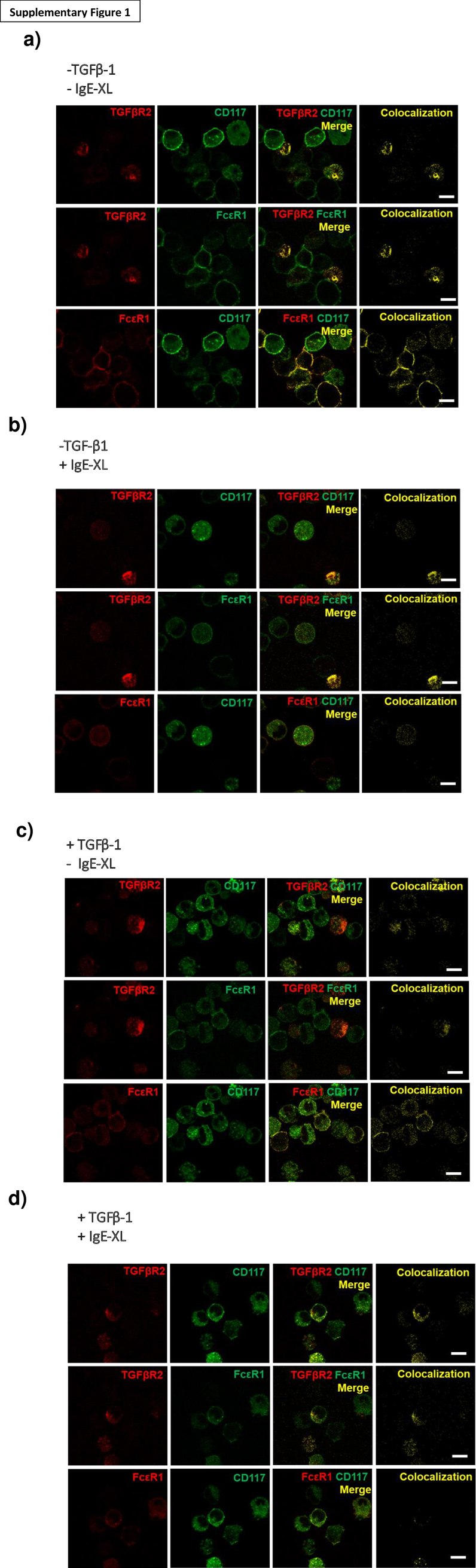

Supplement: S1 Fig — (TIF) [file pone.0207704.s001.tif]
